# PEG3 controls lipogenesis through ACLY

**DOI:** 10.1371/journal.pone.0252354

**Published:** 2021-05-28

**Authors:** Subash Ghimire, Joomyeong Kim

**Affiliations:** Department of Biological Science, Louisiana State University, Baton Rouge, Louisiana, United States of America; University of Illinois, UNITED STATES

## Abstract

*Peg3* (Paternally expressed gene 3) is an imprinted gene encoding a DNA-binding protein that is a well-known transcriptional repressor. Previous studies have shown that the mutant phenotypes of *Peg3* are associated with the over-expression of genes involved in lipid metabolism. In the current study, we investigated four potential downstream genes of *Peg3*, which were identified through ChIP-seq data: *Acly*, *Fasn*, *Idh1*, and *Hmgcr*. *In vivo* binding of PEG3 to the promoter region of these key genes involved in lipogenesis was subsequently confirmed through individual ChIP experiments. We observed the opposite response of *Acly* expression levels against the variable gene dosages of *Peg3*, involving 0x, 1x, and 2x *Peg3*. This suggests the transcriptional repressor role of *Peg3* in the expression levels of *Acly*. Another set of analyses showed a sex-biased response in the expression levels of *Acly*, *Fasn*, and *Idh1* against 0x *Peg3* with higher levels in female and lower levels in male mammary glands. These results overall highlight that *Peg3* may be involved in regulating the expression levels of several key genes in adipogenesis.

## Introduction

Epigenetics is an external modification to DNA that affects gene expression without altering the DNA sequence [1]. Genomic imprinting is an epigenetic phenomenon in which one allele is expressed while the other one is silenced, depending on its parental origin. This unusual expression pattern is achieved through DNA methylation and histone modification [2, 3]. Around 200 imprinted genes are predicted to be present in eutherian mammals and believed to be involved in fetal development and animal behaviors [4]. These imprinted genes are typically clustered in specific chromosomal regions [5]. An imprinted domain usually contains 5 to 10 gene members spanning anywhere from a few hundred kilobases to megabases in length. Imprinted genes are generally regulated through shared cis-regulatory elements, which are referred to as imprinting control regions (ICRs) [6].

Paternally expressed gene 3 (*Peg3*) is located on an evolutionarily conserved imprinted domain on human chromosome 19q13.4/proximal mouse chromosome [7]. This conserved imprinted domain spans a 500-kb genomic region and contains six additional imprinted genes: paternally expressed *Usp29* (ubiquitin-specific hydrolase 29), *APeg3* (antisense *Peg3*), *Zfp264* (zinc finger protein 264) and maternally expressed *Zim1* (zinc finger gene, imprinted 1), *Zim2*, and *Zim3* [8]. PEG3 protein has a KRAB-A (Kruppel-Associated Box A) domain at the N-terminus and C2H2 (Cys2His2)-Kruppel-type zinc finger domains at the C-terminus. The C2H2 zinc finger domain is responsible for DNA binding, while the KRAB-A domain is responsible for the physical interaction and subsequent recruitment of another protein called KAP1 (Kruppel-Associated Protein 1) [9]. KAP1 is a well-known corepressor that interacts with several epigenetic modification proteins, including SETDB1 (histone 3 lysine 9 methyltransferase) and DNMT3A (DNA methyltransferase 3A) [10]. The *Peg3* domain has a 4-kb Peg3-DMR (differentially methylated region), which functions as an ICR [11]. This ICR is the most critical for controlling the transcription and imprinting of the *Peg3* domain [11, 12]. Deletion of this ICR results in global change in the transcriptional levels and also causes the biallelic expression of several adjacent imprinted genes [13, 14]. Another study revealed that an alternative promoter, U1, localized 20-kb upstream of Peg3-DMR, is known to establish DNA methylation on the maternal allele of Peg3-DMR during oogenesis [15]. Deletion of the U1 promoter results in a loss of allele-specific methylation of Peg3-DMR, causing the biallelic expression or double dosage of *Peg3* and *Usp29* [15].

ATP-citrate lyase (ACLY) is a 121-kDa cytosolic enzyme that catalyzes mitochondria-derived citrate and coenzyme A into acetyl-coenzyme A (acetyl-CoA) and oxaloacetate (OAA) by hydrolyzing ATP [16]. *ACLY* is known to link the glucose metabolism to the fatty acid (FA) and the cholesterol synthesis pathways. Cytosolic acetyl-CoA is a building block for the following lipid biosynthetic pathways. First, through fatty acid synthesis, acetyl-CoA is converted to triglyceride for the storage of fat within cytoplasmic lipid droplets. Second, through cholesterogenesis, acetyl-CoA leads to the synthesis of cholesterol and isoprenoids [17]. *ACLY* is overexpressed in several tumors [18–21]. In one of the studies, ACLY production was found to be significantly higher by more than 160-fold in breast carcinoma [22]. Additionally, one of the hallmarks of the tumor is higher levels of *de novo* FA biosynthesis as a result of a significant increase in the expression and activity of several enzymes involved in the FA synthesis pathway [23]. Fatty-acid synthase (FASN), which is involved in *de novo* FA synthesis, is also found to be overexpressed in numerous cancers, including breast, lung, stomach, ovary, colon, and prostate cancers [24]. Furthermore, isocitrate dehydrogenase 1 (IDH1), which catalyzes the reverse reaction of converting α-ketoglutarate to isocitrate in the cytoplasm for the synthesis of body fat and lipids, is responsible for *de novo* lipogenesis and is a major NADPH producer [25–27]. Lastly, several tumors also show an increase in the amount of the enzyme, 3-hydroxy-3-methylglutaryl-CoA reductase (HMGCR), or a lack of feedback mechanism for HMGCR in the mevalonate (cholesterol synthesis) pathway [28].

A previous study involving genome-wide expression analyses revealed that the lipid metabolism is affected in the mutant model of *Peg3* [29]. Another study has shown that *Peg3* mutant mice had an excess of abdominal, subcutaneous, and intra-scapular fat even in low food intake [30]. While this would suggest that *Peg3* may be responsible for the regulation of fat metabolism, there are still knowledge gaps in terms of how this might be accomplished. In the current study, we identified *Acly*, *Idh1*, *Fasn*, and *Hmgcr* as potential downstream genes from ChIP-seq analysis. According to the results, the promoter of these genes is bound by *Peg3* and the variable dosages of *Peg3* affect the expression levels of these genes. This suggests that PEG3 may play a role in regulating the lipogenesis pathway via ACLY.

## Results

### Variable gene dosages of *Peg3* (0x, 1x, and 2x)

**Fig 1:**
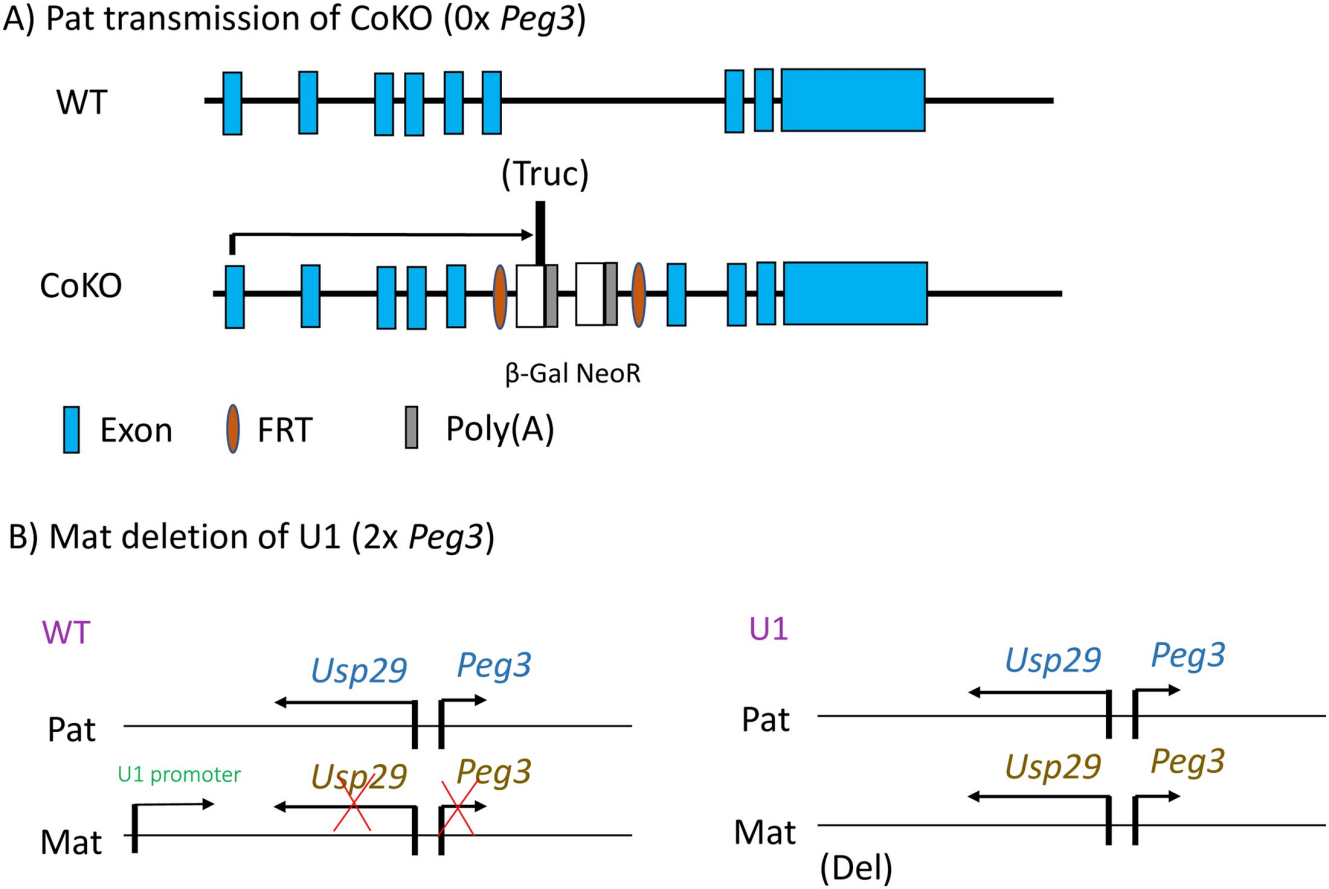
Genomic structure of CoKO and U1 mutant alleles of the *Peg3* locus. (A) Schematic representation of WT and CoKO allele of the mouse *Peg3* locus. Exons of *Peg3* are indicated with blue boxes. The 7.1-kb insertion cassette contains promoterless galactosidase (β-Gal) and human β-actin promoter-driven neomycin resistance gene (NeoR), which are indicated with white boxes. The Poly(A) tails associated with these genes are indicated with grey boxes. This expression cassette is flanked by brown oval-shaped FRT boxes. The Poly(A) tails are accountable for the truncation of *Peg3* expression, thus the 0x dosage of *Peg3*. (B) Schematic representation of WT and U1 alleles. The blue color indicates the genes associated with the paternal allele and the orange color represents the genes associated with the maternal allele. Maternal deletion of an alternative promoter, U1, causes the expression of *Peg3* and *Usp29* from the maternal allele, subsequently responsible for the expression levels of 2x dosage of *Peg3*.

For the current study, we used the following two mutant alleles with variable dosages of *Peg3* [29, 31]. The first mutant allele is referred to as CoKO (Conditional KnockOut-ready). This model was constructed to truncate transcription of *Peg3* by inserting two poly(A) signals as a part of an expression cassette containing a promoterless β-galactosidase gene and the neomycin resistance gene driven by the human β-actin promoter within the 5th intron of *Peg3* (Fig 1A) [29]. With the paternal transmission (+/-^P^), the gene dosage of *Peg3* is almost zero in heterozygous individuals, thus the 0x dosage of *Peg3*. Conversely, with the maternal transmission (-^M^/+), the dosage is similar to the WT, the 1x dosage of *Peg3*, because it is already silenced by DNA methylation [29]. The second mutant allele is U1 (2x *Peg3* dosage), which has a 1-kb deletion of the genomic region containing an alternative U1 promoter located 20-kb upstream of the Peg3-DMR region (Fig 1B). Deletion of the U1 promoter results in the biallelic expression of *Peg3* and *Usp29* due to the absence of oocyte-driven DNA methylation on the Peg3-DMR [15]. With the maternal transmission of the U1 allele, the gene dosage of *Peg3* is almost double in F1 heterozygote, thus the 2x dosage of *Peg3* [32]. On the other hand, with the paternal transmission, there is no difference in the gene dosages of *Peg3* compared to WT. For the current study, we performed the following set of breeding experiments to harvest the tissues with variable dosages of *Peg3*. For the 0x dosage of *Peg3*, WT females were crossed with males heterozygous for *Peg3*^*CoKO*/+^ to achieve the paternal transmission of the CoKO allele. We harvested two biological replicates of 14.5-dpc (days post-coitum) embryos of each sex with WT and CoKO genotypes. Likewise, the mammary gland was extracted from two 1.5-month-old virgin adults of each sex with WT and CoKO. For the 2x dosage of *Peg3*, WT males were crossed with females heterozygous for *Peg3*^*U1*/+^. Similar to CoKO breeding, we harvested two biological sets of 14.5-dpc embryos and 1.5-month-old individuals of both sexes with WT and U1 genotypes, from which the mammary gland was harvested.

### Genome-wide scanning and pathway analysis of *Peg3* downstream genes

We analyzed ChIP-seq results, which were derived from mouse embryonic fibroblast cells (MEFs) from WT and CoKO samples [31]. Raw sequence reads were analyzed using the bioinformatic pipeline involving a peak prediction program, MACS2 [33]. This program identified around 16 downstream genes of *Peg3* with a significantly high average p-value being around 25 [34]. However, MACS2 only predicted a very small number of peaks due to its highly stringent peak selection parameters. We further extended this analysis by manually scanning the ChIP-seq peaks across the whole mouse genome. We identified peaks with a minimum threshold p-value of 10 but with no peak on the *Peg3* mutant (CoKO) samples, which were used to filter background noise. A total set of 1073 genes were identified from this analysis (S1 File). The majority of the peaks were overlapped with the promoter regions of the individual genes. We then performed pathway analysis, which revealed that some pathways have a greater number of downstream targets of *Peg3* than the other pathways. Interestingly, most of these are well-studied pathways: cancer pathway (32 out of 328 genes), p53 signaling pathway (8 out of 69 genes), MAPK signaling pathway (21 out of 267 genes), WNT signaling pathway (12 out of 151 genes), and Jak-STAT signaling pathway (9 out of 155 genes) (S1 Fig). These results go along with the previous *Peg3* studies revealing its connection to cancer, p53, Wnt, and Jak-STAT pathways [35–38].

**Fig 2:**
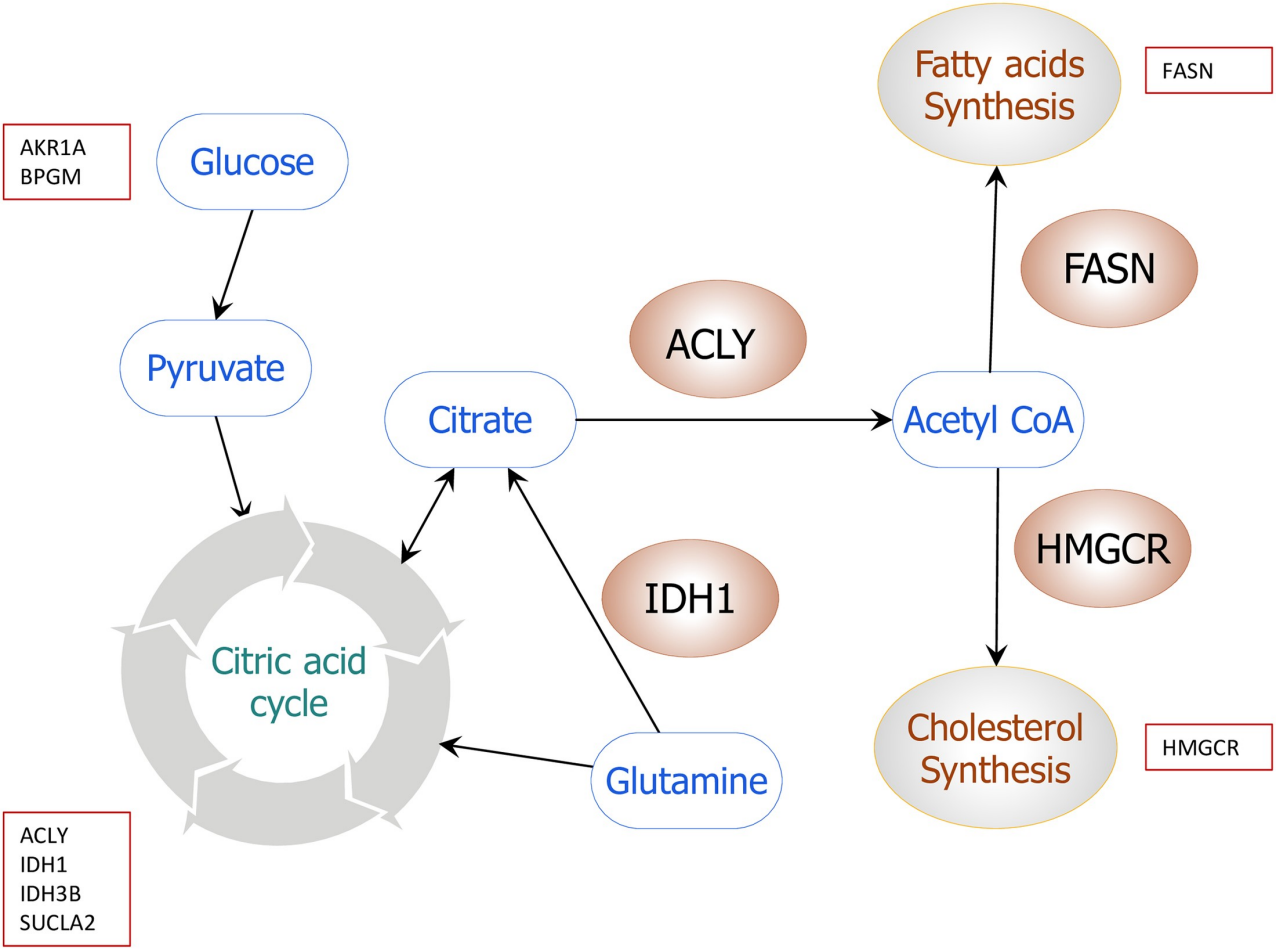
*Peg3* downstream genes involved in major metabolic pathways. The schematic figure shows the combination of several metabolic pathways from glycolysis to lipogenesis (fatty acid synthesis and mevalonate pathway). Glucose-derived citrate is converted to acetyl-CoA by ATP-citrate lyase (ACLY), which is used as a precursor in fatty acid and the mevalonate synthesis pathways. This current study identified 1073 genes as the downstream genes of *Peg3* from genome-wide scanning of ChIP-seq peaks. More than 30 pathways were examined to test if there were any overlapping genes between these pathways and the genes identified from ChIP-seq analysis using the Kegg pathway database (https://www.kegg.jp/kegg/). Initial analysis identified several key genes critical for lipogenesis, including *Acly*, *Idh1*, *Fasn*, and *Hmgcr*.

*Peg3* is also known to be closely associated with several metabolic pathways, including lipid metabolism based on the mutant phenotypes of *Peg3* [29, 30, 32]. Thus, we further examined whether there is an overlap between these potential downstream genes of *Peg3* and the genes involved in the metabolic pathway. Around 171 downstream genes were found to be overlapped out of the total set of 1073 genes when we analyzed more than 30 key metabolic pathways. Among these metabolic pathways, the lipogenesis pathway was of particular interest based on the following reasons. Several key genes critical for the lipogenesis were found to be potential downstream genes of *Peg3*: *Acly* (ATP-citrate lyase), *Fasn* (Fatty-acid synthase), *Idh1* (isocitrate dehydrogenase 1), and *Hmgcr* (3-hydroxy-3-methylglutaryl-CoA reductase). In particular, these genes are also positioned at the entry point of each synthesis pathway (Fig 2). ACLY links the glycolysis and TCA cycle to the fatty acid and the cholesterol synthesis pathway by providing acetyl-CoA [17]. Similarly, IDH1 mediates reductive carboxylation of α-ketoglutarate to isocitrate for the synthesis of lipids. Overexpression of *Idh1* is associated with an increase in body weight, fat mass, serum cholesterol, and triacylglycerols [39]. FASN is a key enzyme in the fatty acid synthesis pathways, β-oxidation, and lipid modification of cellular proteins [23]. Lastly, HMGCR is a critical enzyme involved in the first and rate-limiting step in the mevalonate pathway. Inhibition of HMGCR in normal cells triggers a robust homeostatic feedback response that ensures the cells to upregulate and restore the mevalonate pathway [40]. Given the close connection of *Peg3* to lipogenesis, we further analyzed these four genes as described below.

### *In vivo* binding of PEG3 to the genes involved in lipogenesis

**Fig 3:**
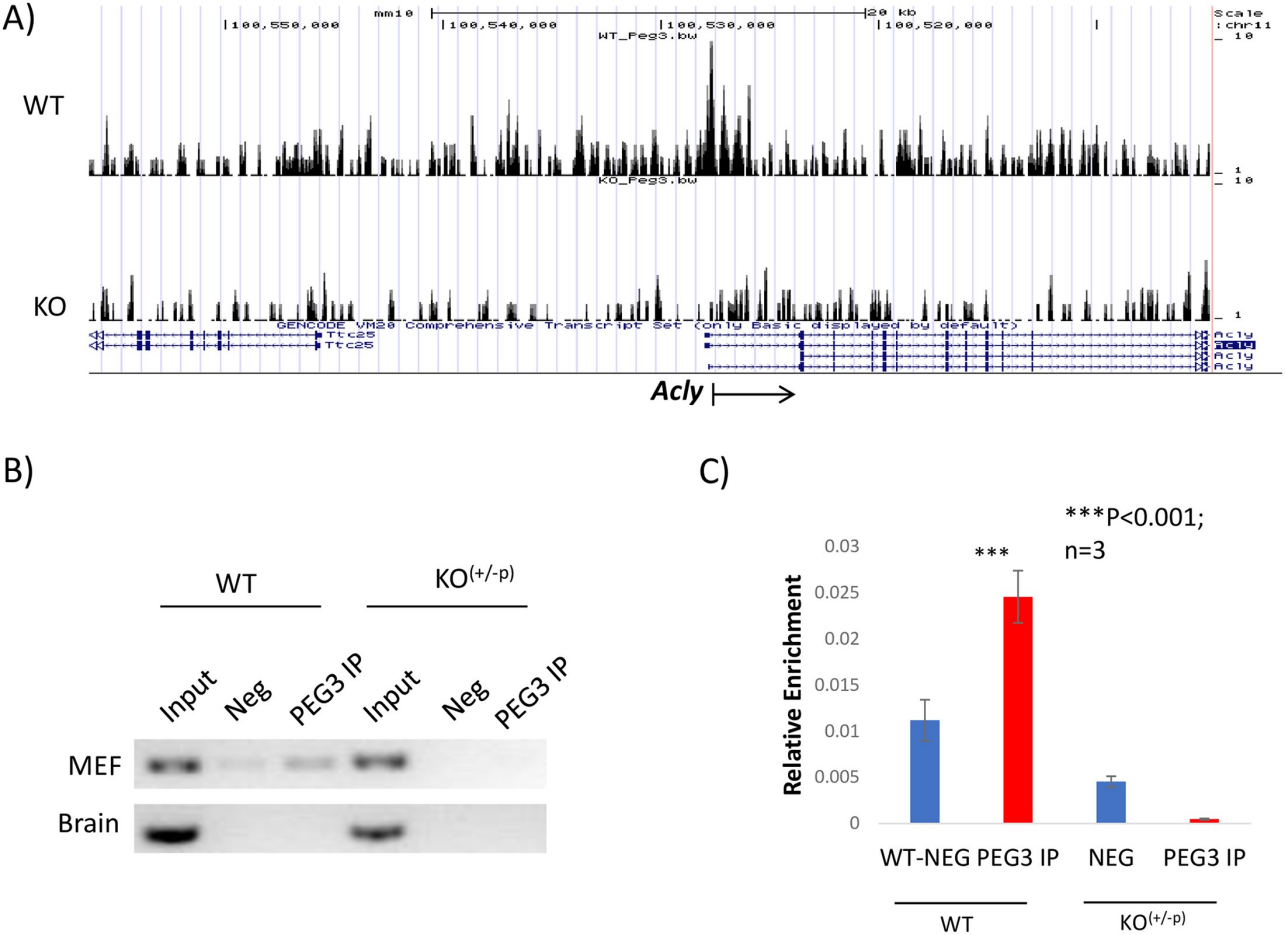
*In vivo* binding of PEG3 to the promoter of *Acly*. (A) ChIP-seq results from the 80-kb genomic intervals encompassing *Acly*. The top panel is from WT-MEF cells and the bottom panel is from KO-MEF cells. Statistical p-values are indicated on the Y-axis with the maximum value being 10, whereas relative genomic positions are on the X-axis. An arrow represents the transcriptional direction of *Acly*. (B) Individual ChIP experiment confirming the *in vivo* binding of PEG3 to the promoter regions of *Acly*. Chromatins were prepared from the 14.5-dpc embryos and neonatal brain of WT and CoKO. Template for PCR reaction was the immunoprecipitated DNA either from WT or KO-MEF cells with the anti-PEG3 antibody. In addition, the Input and Negative (Neg) DNA was also included in this PCR amplification. The Neg control contained the DNA from the ChIP experiment without the antibody. (C) Quantitative PCR results from the immunoprecipitated DNA with the anti-PEG3 antibody. The red and blue bars indicate the relative enrichment levels of PEG3 IP and Neg compared to the Input in the promoter regions of *Acly*. Relative enrichment values are indicated on the Y-axis for each sample. All the analyses were performed in triplicates. The statistical significance of the observed difference between PEG3 IP and Neg was tested with a student t-test (*, p-value <0.05 and ***, p-value <0.0001).

The potential downstream genes of *Peg3* involved in lipogenesis were further confirmed by individual ChIP experiments. Two sets of chromatins were prepared from MEF cells and neonatal brains with each set representing WT and CoKO samples. Each ChIP derived three individual DNA: Input, Negative control (Neg), and the immunoprecipitated DNA with anti-PEG3 antibody (PEG3 IP). Individual immunoprecipitated DNA was analyzed with a fixed number of PCR cycles (38 cycles) as well as a quantitative PCR (Fig 3). Four individual primer sets were designed and used to target the promoter regions of the potential downstream genes. In the case of *Acly*, this analysis showed the detectable levels of enrichment in WT MEF but no enrichment levels in CoKO MEF, confirming the *in vivo* binding of PEG3 to the promoter of *Acly* (Fig 3B). Results from qPCR also corroborated this result as the enrichment levels were almost 5-folds higher than those of CoKO MEF (Fig 3C). However, no detectable levels of the enrichment of DNA were observed for both WT and CoKO in the brain (Fig 3B). We were also able to confirm the binding of PEG3 to *Idh1* in both the MEF cells and the brain (S2 Fig). Furthermore, we confirmed the *in vivo* binding of PEG3 to *Hmgcr* and *Fasn* in the MEF cells but not on the brain (S3 and S4 Figs). Overall, this series of analyses confirm the binding of PEG3 to *Acly*, *Fasn*, and *Hmgcr* in MEF and *Idh1* in both MEF and neonatal brain.

### Expression level changes of *Acly* in response to the variable dosages of *Peg3*

To examine the functional outcomes of different dosages of *Peg3* on the expression levels of *Acly*, a series of qRT-PCR were performed. According to the previous studies, higher expression levels of *Peg3* were observed in embryos, placenta, hypothalamus, and mammary gland [7]. Thus, we chose embryos as the first target tissue. Also, among these tissues, the mammary gland was noteworthy for the following two reasons. Firstly, the mutant phenotypes of *Peg3* are associated with a defect in milk provision and involve the mammary gland [41, 42]. Secondly, the mammary gland produces milk that has a high percentage of fatty acid globules [43]. These reasons prompted us to pursue the mammary gland as the second target tissue for the following examinations.

**Fig 4:**
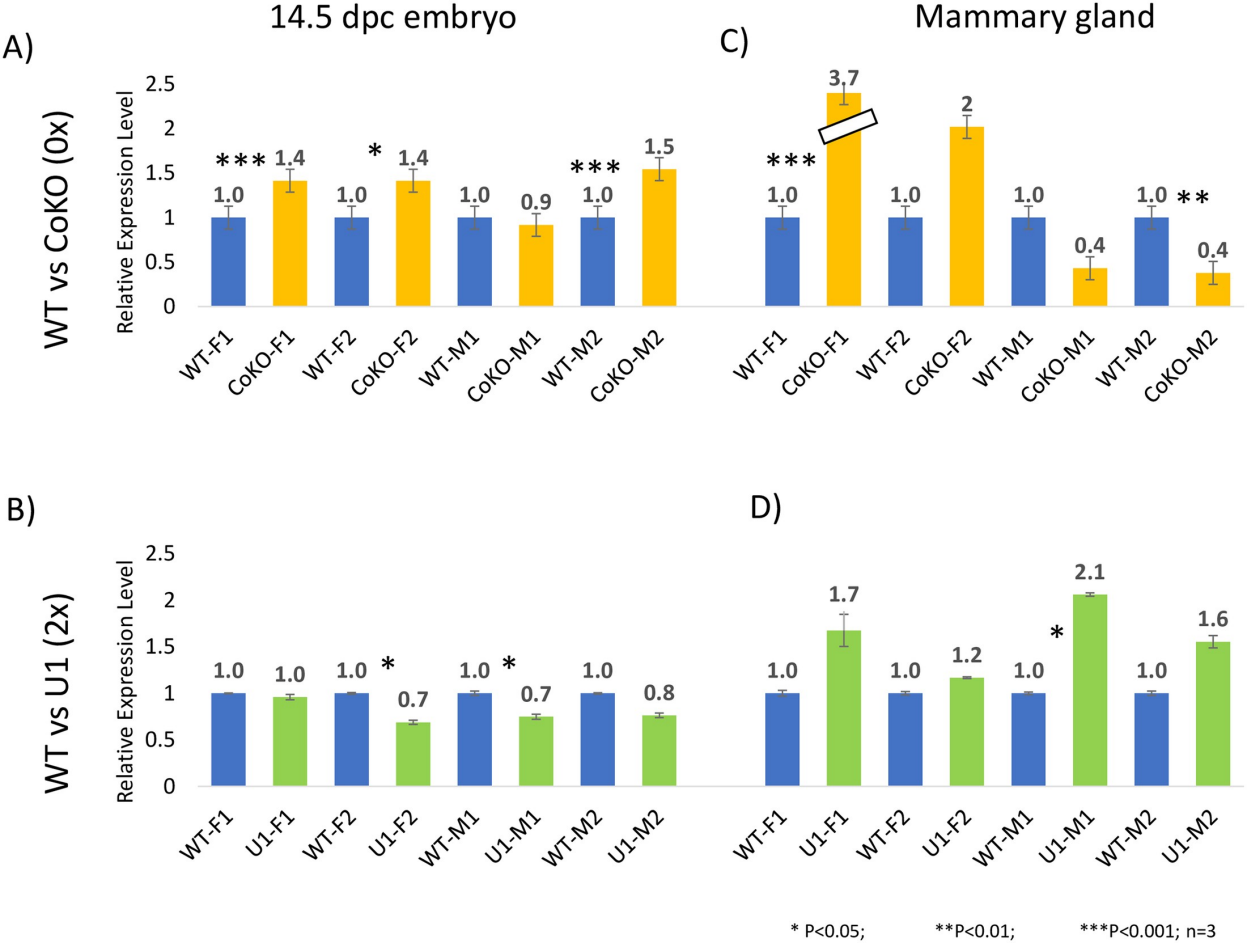
Expression level changes of *Acly* against the 0x and 2x dosage of *Peg3*. Expression levels of *Acly* were measured using a series of qRT-PCR. This set of analyses was performed using two biological replicates for both the embryo and the mammary gland set of males (M1 and M2) and females (F1 and F2). The total RNA was isolated from the embryos and the mammary glands with WT (1x), CoKO (0x), and U1 (2x) dosage of *Peg3*. The expression level differences in the 14.5-dpc embryos were summarized in the first column (A, B) and the adult mammary glands in the second column (C, D). Individual gene expression levels were first normalized to *Gapdh* levels and then normalized values were further compared between WT and the mutants (CoKO or U1). Subsequent relative levels are presented in a graph with the average values and standard deviations. The statistical significance of the observed difference between WT and mutants was tested with a student t-test (*, p-value <0.05 and ***, p-value <0.0001). The corresponding p-values are listed in the text.

We examined two biological replicates of 14.5-dpc embryos and the mammary gland of 1.5-month-old adult virgin mice of each sex. F1 and F2 represent two biological female samples, whereas M1 and M2 represent two biological male samples. The total RNA was isolated from these samples, used for cDNA synthesis, and finally analyzed with qRT-PCR. The expression levels were compared among the WT (1x), CoKO (0x), and U1 (2x) samples. *Gapdh* was used to normalize the expression levels of each gene before comparing WT and U1/CoKO. The results are summarized as follows. First, for 14.5-dpc embryo samples, the expression levels in CoKO increased by about 1.4-fold in 3 out of 4 embryos. These observed upregulations were statistically significant with the p-value being lower than 0.03 (Fig 4A). In the case of U1 samples, however, there was an opposite effect, with U1 showing about a 30% decrease in the expression levels of *Acly* in embryos, with 2 out of 4 samples being statistically significant (p = 0.02; Fig 4B). Second, for adult mammary glands, the expression levels of *Acly* in both female CoKO samples were around 2-fold higher than WT samples, whereas the levels in male CoKO samples were lower by 60% than WT samples, showing sex-specific up and down-regulation in females and males respectively (p = 0.004–1; Fig 4C). In contrast, for U1 samples, there was a 1.2–2.1 fold increase in the expression levels of *Acly* in both female and male adult mammary glands (p = 0.02–0.09; Fig 4D). Overall, this series of expression analyses provides two immediate conclusions. First, in embryo, *Acly* responded oppositely against *Peg3* dosage, which is consistent with the predicted function of *Peg3* as a repressor [34, 44, 45]. Second, sex-biased results were observed, especially in the adult mammary gland, as the expression levels of *Acly* was up in female but down in male, albeit, only in CoKO samples.

### Expression level changes of the downstream genes in the adult mammary gland

**Fig 5:**
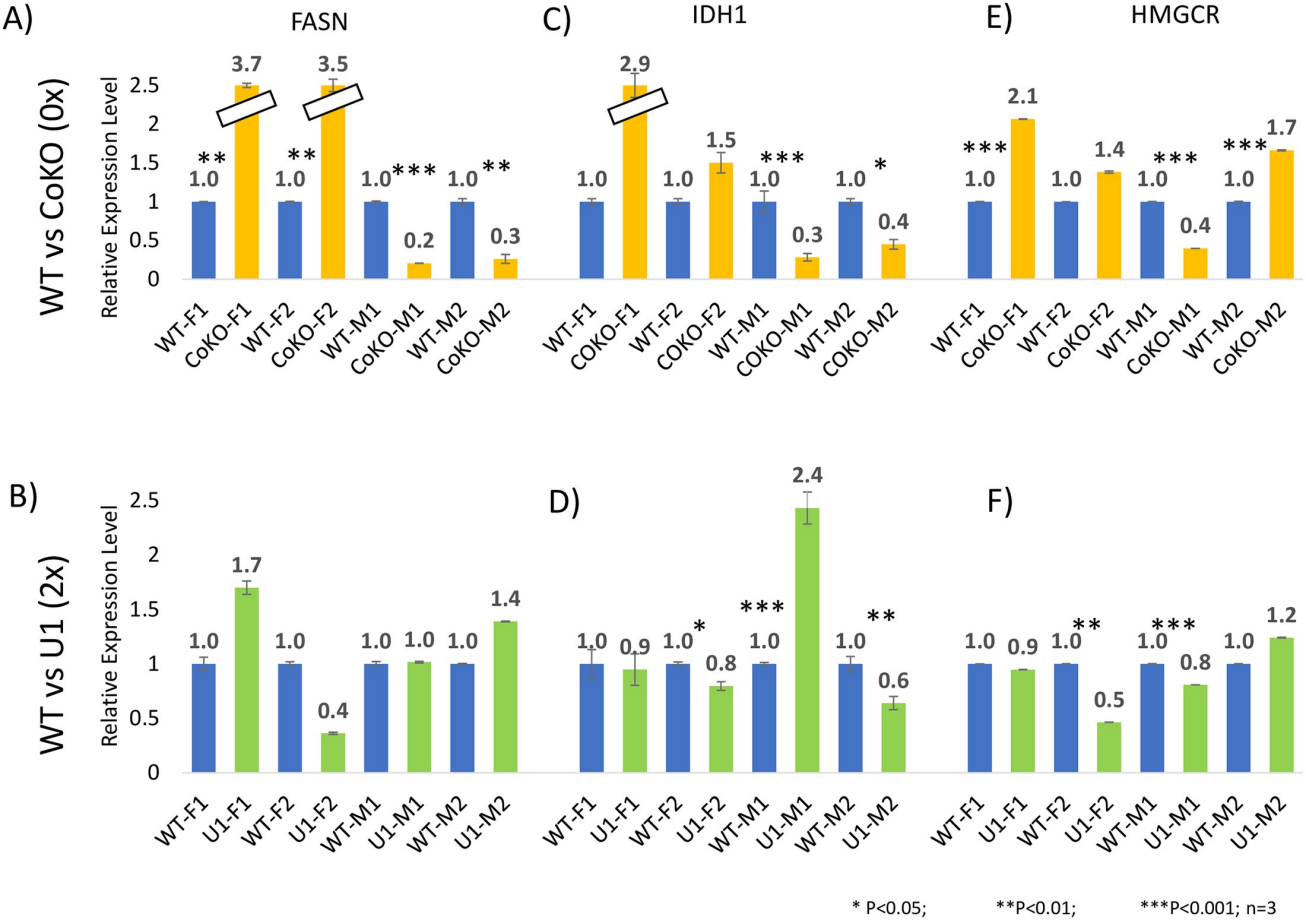
Expression level changes of *Fasn*, *Idh1*, and *Hmgcr* in adult mammary gland. A series of qPCR assays were performed to measure the expression levels of *Fasn*, *Idh1*, and *Hmgcr* between WT and CoKO/U1. For these analyses, two males (M1 and M2) and two females (F1 and F2) were used. Total RNA was isolated from the adult mammary glands, which was subjected to cDNA synthesis. A qRT-PCR was then performed. *Gapdh* was used to normalize the expression levels of each gene before comparing between WT and U1/CoKO. The results sets were summarized with the average values and standard deviations. The statistical significance is indicated in the following student’s t-test (*, p-value <0.05 and ***, p-value <0.0001). The corresponding p-values are listed in the text.

We performed another series of expression analysis with a set of additional downstream genes, *Fasn*, *Idh1*, and *Hmgcr*. A similar series of qRT-PCR analyses were performed using total RNA isolated from the adult mammary gland as described above. The results are summarized as follows. First, for *Fasn*, the expression levels in the CoKO females were about 3.5-fold greater than WT samples, whereas the expression levels in the CoKO males were 70% lower than WT (p<0.003; Fig 5A). In the case of U1 samples, there were no significant changes except the U1-M1 sample, which showed a 60% decrease in expression levels (p = 0.01–0.9; Fig 5B). Second, regarding *Idh1*, there was about a 1.5–2.9 fold increase in expression levels in the CoKO females whereas expression levels significantly decreased by around 60% in CoKO male samples (p = 0.006–0.39; Fig 5C). However, for the U1 sample, there was a decrease in expression levels in both sexes except the U1-F1 sample (p = 0.04–0.9; Fig 5D). The observed expression levels of both *Fasn* and *Idh1* were similar to the pattern seen in *Acly*, showing sex-specific up and down-regulation of expression levels in the females and the males of CoKO respectively. Finally, for *Hmgcr*, the expression levels were 1.4–2.1 fold higher in CoKO females (p = 0.0059–0.66). Results from male CoKO samples, nonetheless, were inconclusive as two biological samples portrayed contrasting expression levels (Fig 5E). Unlike CoKO, the expression levels of the U1 samples decreased in females (p = 0.004–0.2) and were similarly inconclusive in males (Fig 5F). It is prudent to mention that the variations were observed in the expression levels among the biological replicates. This might have been due to unknown variations in tissue samples, individual mice, and litter numbers. Together, these results indicate sex-specific responses of *Fasn* and *Idh1*, especially in CoKO samples, but little or no responses in U1 samples.

## Discussion

In this study, we characterized four downstream genes of *Peg3* involved in lipogenesis: *Acly*, *Idh1*, *Hmgcr*, and *Fasn*, which were identified through analyzing previous ChIP-seq data [34]. Through individual ChIP experiments, we further confirmed the *in vivo* binding of PEG3 to the promoter regions of *Acly*, *Fasn*, *Idh1*, and *Hmgcr*. Expression analyses using two mutant mouse models, CoKO (0x *Peg3*) and U1(2x *Peg3*), demonstrated the opposite response of *Acly* expression levels against the dosage of *Peg3* in embryos, suggesting *Peg3* as a transcriptional repressor for the expression levels of *Acly*. On the other hand, the analyses using the mammary gland showed a sex-specific opposite response of the expression levels of *Acly*, *Fasn*, and *Idh1* against 0x *Peg3*, with higher levels in females and lower levels in males as compared to the wildtypes (Figs 4 and 5). Overall, this study suggests that *Peg3* is involved in regulating the expression levels of key genes in adipogenesis.

**Fig 6:**
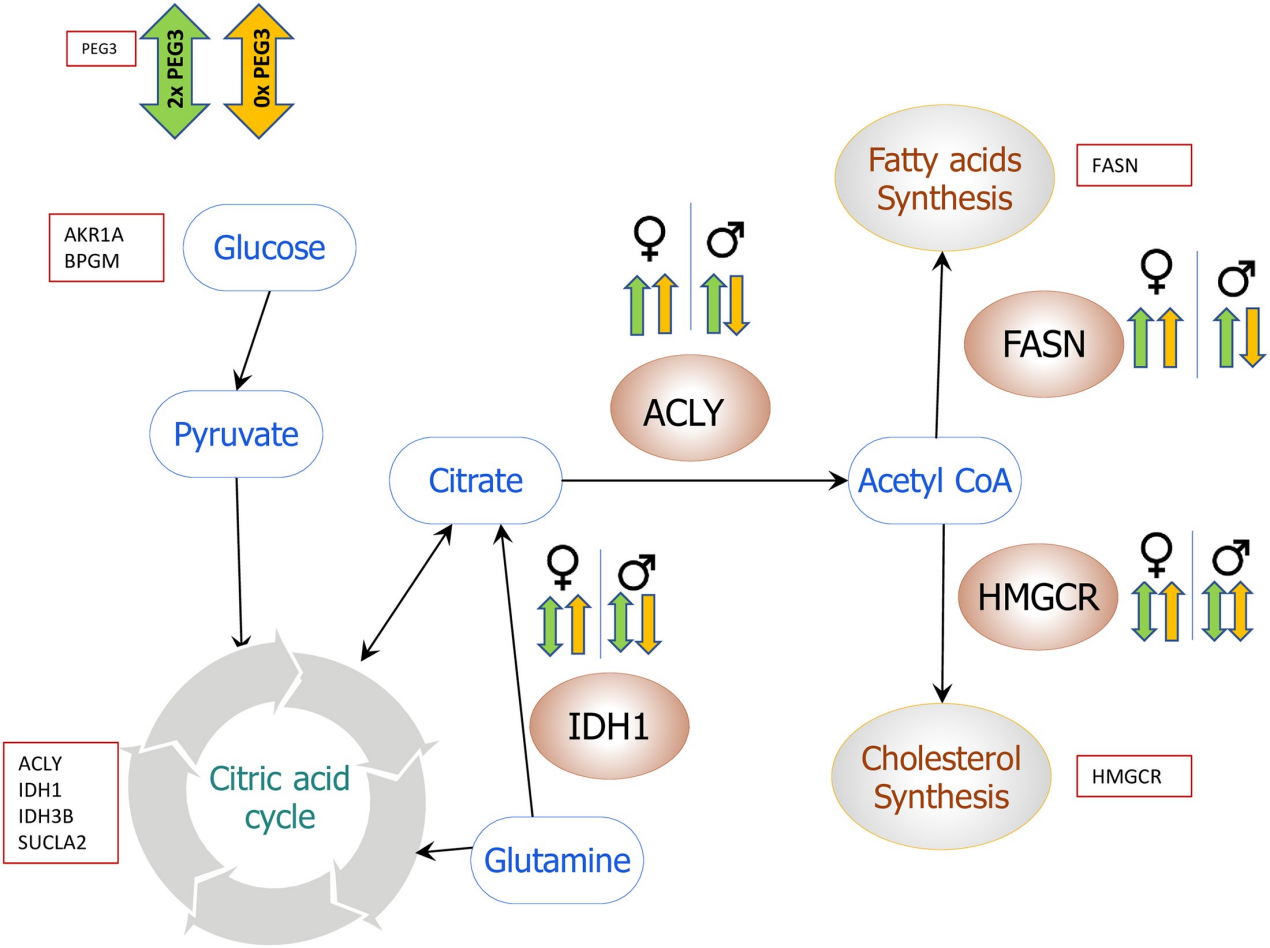
*Peg3*’s potential control on lipogenesis through *Acly*. Schematic representation of the potential downstream genes of *Peg3* and their associated metabolic and lipogenesis pathways. Red color genes, *Fasn*, *Idh1*, and *Hmgcr*, are potential downstream genes predicted from the ChIP-seq analysis. All of these genes are involved in either regulating *Acly* or fatty acid and mevalonate pathways. With the variable dosages of *Peg3*, changes were observed in the expression levels of these genes, which are indicated with vertical arrows. Yellow arrows indicate 0x dosage of *Peg3* and green arrows represent the 2x dosages of *Peg3*. Sex-specific response in the expression levels of *Acly*, *Fasn*, and *Idh1* in the adult mammary gland was also observed in CoKO (0x *Peg3*). This suggests that *Peg3* may play a role in lipogenesis by regulating the expression levels of *Acly* and several key genes.

*Peg3* appears to be a DNA-binding regulator for *Acly* as *Peg3* is shown to bind to the promoter region of *Acly* (Fig 3). The *Peg3* dosages also affected the expression levels of *Acly* in embryos (Figs 3, 4A and 4B). These results comply with the previous studies showing the DNA-binding capability of PEG3 and controlling the expression levels of several downstream genes, including *Oxtr*, *H19*, *Msl1*, and *Msl3* [34, 44, 46, 47]. In the mammary gland, *Peg3* dosage appeared to be associated with the expression level of the key gene, *Acly*, connecting the glucose metabolism and lipid synthesis (Fig 6). It has already been shown that the loss-of-function mutation on *Peg3* contributes mice to have a smaller body size but with a higher proportion of adipocytes than the wildtypes and the upregulation of the genes involved in lipid metabolism [29, 30]. Moreover, epigenetic silencing of *PEG3* is frequently associated with several cancers, suggesting a tumor suppression function of *PEG3*. One study also reported the reduced expressions of *PEG3* in 18 different types of cancers in humans [48]. Additionally, the loss of *PEG3* expression is connected to ovarian, breast, and cervical cancer [49–51]. Furthermore, a significant upregulation of *ACLY* expression has been associated with several tumors, including lung, prostate, bladder, breast, liver, stomach, and colon tumors [18–21]. Interestingly, tumor progression is associated with an increase in lipid synthesis, and thus *ACLY* tends to be overexpressed in such cancer cells to accelerate lipid synthesis and tumor progression [52]. ACLY has already been identified as a potential molecular target for cancer therapy, as *de novo* fatty acid synthesis occurs at very high rates in tumor tissues [17]. This aligns with our finding that the silencing of *PEG3* might upregulate the expression levels of *ACLY*, which might be required for the high demand of lipids for the rapidly dividing cancer cells. Overall, this suggests that *Peg3* is the upstream regulator of *Acly* by binding to the promoter and affecting the expression levels of *Acly*.

We observed a sex-specific opposite response in the expression levels of *Acly*, *Idh1*, and *Fasn* in the adult mammary gland in CoKO (0x *Peg3*), showing upregulation in females and downregulation in males as compared to those of WT littermates (Figs 4 and 5). It has already been shown that *Peg3* and *Acly* are sexually dimorphic in their expression levels [13, 53]. Therefore, the expression levels of *Peg3* may be regulated by sex-specific hormones. Consistent with this, our meta-analysis revealed that one of the potential enhancers of *Peg3*, ECR17 (Evolutionarily Conserved Region), has a binding site for the male-specific sex hormone, androgen [54]. Similarly, the mammary gland, our tissue of interest, is fully developed in females during puberty in response to the release of estrogen, which itself is a sex-specific hormone [55, 56]. Therefore, lipid and glucose metabolisms in the mammary gland are likely sex-specific processes [57]. It is also important to note that the sex-specific response was only observed in the mammary glands but not in the embryos. This discrepancy can be explained by the fact that the mammary gland is a highly differentiated and specialized tissue with specific functions, whereas the embryo comprises the whole body. All these factors combined might have contributed to the sex-specific results as different sexes were behaving differently to the dosages of *Peg3*, in particular 0x dosage of *Peg3*.

Reduced gene dosage of *Peg3*, 0x *Peg3* (CoKO), appears to contribute more significant fluctuation to the expression levels of *Acly*, *Fasn*, and *Idh1* compared to double dosage of *Peg3*, 2x *Peg3* (U1) in mammary glands (Figs 4 and 5). We expect two possibilities that might contribute to the discrepancy. First, ACLY is a very critical enzyme, which provides acetyl groups for histone acetylation/gene regulation and lipid synthesis. Thus, several additional genes or regulatory factors may regulate the expression of *Acly* [58, 59]. By contrast, *Peg3*’s role in the regulation of *Acly* expression might be minimal or indirect. This might be a reason for the observed discrepancy. Second, *Acly*, *Fasn*, and *Idh1* are the major genes for metabolic pathways and their expression levels are, in general, already high in the cells and also critical for the survival of organisms [60–62]. As a result, the expression levels of these genes may be tolerable only in one of the two mutant models; the upregulation may be tolerable in CoKO, whereas the downregulation may be intolerable in U1. As a consequence, we might be able to observe the upregulation from the surviving CoKO model, but not from the downregulation from the U1 model. The second scenario seems more plausible since our results showed these genes to be immediate downstream targets of *Peg3* with individual ChIP experiments demonstrating the *in vivo* binding of PEG3 to the promoter regions of *Acly* and *Idh1*.

Lastly, our result showed some degrees of individual variation for both male and female samples. We repeated the expression level analyses several times to minimize the human error as well as to increase the confidence in the results. Since we are dealing with the enzymes and the tissues that are highly modulated mainly through hormonal response, the multitude of subtle factors that could potentially affect the outcome cannot be discredited. However, considering all the hormonal, metabolic, and litter number variation, our results look promising as there is a possible role of *PEG3* in adipogenesis via *ACLY* when the individual is subjected to *PEG3* dosage variation. This might also open the possibility for potential drug design for the tumors involving defective *ACLY* by targeting *PEG3*.

## Materials and methods

### Ethics statement

All the experiments related to mice have been conducted in conformance with the National Institutes of Health guidelines for the care and use of animals. Furthermore, these experiments were also assisted by Louisiana State University Institutional Animal Care and Use Committee (IACUC), protocol 19–079.

### Mouse breeding

The mice used for this experiment were C57BL/6J strain housed on a 12–12 dark-light cycle under a constant temperature of 70°F and 50% humidity at the DLAM (Division of Lab Animal Medicine) at LSU. Two mutant strains of *Peg3* were used, which have been previously characterized: the CoKO strain with the modification within its 5th intron of *Peg3* and the U1 strain with the 1-kb deletion of the alternative U1 promoter [29, 63]. All the mice were euthanized by CO_2_ asphyxiation under the rules and regulations set forth by the IACUC. Breeding experiments were performed through the crossing of males *Peg3*^*CoKO*/+^ with WT females and females *Peg3*^*U1*/+^ with WT males. For the embryos, individual embryos were isolated along with placentas from pregnant females. The latter was used for genotyping and sex determination of the associated embryo. Embryos were then snap-frozen in liquid nitrogen for future experiments. Likewise, adult mice were also genotyped by using genomic DNA obtained from clipped ears. These tissues were incubated in lysis buffer (0.1 M Tris-Cl, pH 8.8, 5 mM EDTA, pH 8.0, 0.2% SDS, 0.2 M NaCl, 20 μg/ml Proteinase K) overnight at 55°C. For genotyping, the following set of primers were utilized: for the CoKO allele, Peg3-5arm (5′-CCCTCAGCAGAGCTGTTTCCTGCC-3′) and LAR3 (5′-CAACGGGTTCTTCTGTTAGTCC-3′); for the U1 allele, P1 (5’-TAGCAAGGGAGAGGGCCTAG-3’), P2 (5’-GGAAGCCTCCATCCGTTTGT-3’), and P3 (5’-AGCACAGCTAGAAATACACAGA-3’). Coupled with physical appearance, the sex of each pup was also determined via PCR using a forward and reverse set of primers: mSry-F (5’-GTCCCGTGGTGAGAGGCACAAG-3’) and mSry-R (5’-GCAGCTCTACTCCAGTCTTGCC-3’).

### RT-qPCR

Trizol isolation kit (Invitrogen) was used to isolate total RNA from the extracted tissues, either embryo or adult mammary gland of mice. Random hexamer was added to the isolated RNA (1 ug), which was then followed by the addition of M-MuLV reverse transcriptase (New England Cat. No. M0253S). For each gene tested, cDNA was used as a template for quantitative PCR. The SsoAdvanced^™^ Universal SYBR^®^ Green Supermix (Bio-Rad) was used for qRT-PCR analysis using the ViiA^™^ 7 Real-Time PCR system (Life Technologies). All qRT-PCR analyses were conducted for 40 cycles under standard PCR conditions. Expression levels of the genes were normalized to internal control, *Gapdh*, and analyzed further based on the threshold (Ct) value. The Ct value of a technical replicate of a particular gene was subtracted from the average Ct value of the internal control (*Gapdh*) for that gene to calculate the ΔCt value. The fold difference was calculated for each replicate by raising 2 to the ΔCt powers [64]. The average and standard deviation of fold differences of each sample were then plotted. For statistical comparison, a one-tailed Student’s t-test was used on the samples. More detailed information on each of the primer sequences is available in the (S2 File).

### ChIP and ChIP-seq analyses

Chromatins were prepared from MEFs and neonatal brains according to the methods previously described [11]. Briefly, one percent formaldehyde was used to crosslink homogenized cell samples and then lysed with the lysis buffer containing protease inhibitor cocktail (Millipore, Cat. No. 539131). The lysed sample was sonicated to derive 300 to 500 bp sizes of DNA fragments, which were then immunoprecipitated against the commercial polyclonal anti-PEG3 antibody (Abcam, Cat. No. ab99252). Protein A/G PLUS-Agarose beads (Santa Cruz, Cat. No. sd-2003) were used to pull down the protein complexes. Finally, the isolated protein complexes were de-crosslinked and treated with phenol-chloroform. The purified DNA was dissolved in 100 ul of TE buffer for later use. For ChIP-seq analysis, two different samples of MEF cells (WT and CoKO) were used. A combination of these two samples with the corresponding input DNA were utilized for the ChIP-seq library construction following the manufacturer’s protocol (Illumina FC4014003). Bowtie2 was used to map all of the initial raw sequences, which were around 35 million reads per sample, to the mouse genome sequence [65]. Next, we processed the sam files obtained from mapping with Bowtie2 to the bigwig files. Bigwig files were uploaded to the UCSC genome browser for peak visualization. A gene list was created by manually inspecting the ChIP-seq peaks with a minimum threshold p-value of 10 throughout the mouse genome. The obtained gene list was used for pathway analyses using the Kegg pathway database (https://www.kegg.jp/kegg/). Overlapped sets of genes between the identified genes from ChIP-seq data and various pathways were identified using Venny software (https://bioinfogp.cnb.csic.es/tools/venny/). All the final output files are available in the (S1–S3 Files).

## Supporting information

S1 Fig List of pathways with potential downstream genes of *Peg3*. Thirty pathways were examined to find the overlapping genes between the gene list containing the downstream genes of *Peg3* and the given pathway. Manual inspection of ChIP-seq results revealed 1073 downstream genes of *Peg3*. Out of these genes, several of them belong to already well-studied pathways as shown in the figure.
(TIF)

S2 Fig *In vivo* binding of PEG3 to the promoter of *Idh1*. The binding of PEG3 to *Idh1* was examined using ChIP-seq and individual ChIP experiments. (A) Forty-one kb genomic regions surrounding *Idh1* from ChIP-seq data. The peak was observed on the promoter region of *Idh1* in WT sample. (B) *In vivo* binding of PEG3 to *Idh1* in the MEF and brain. Individual ChIP experiment confirmed the binding of PEG3 to the promoter region of *Idh1* in both MEF and brain samples.
(TIF)

S3 Fig *In vivo* binding of PEG3 to the promoter of *Hmgcr*. ChIP-seq and individual ChIP experiments were performed to assess the binding of PEG3 to *Hmgcr*. (A) Thirty-two kb genomic regions encompassing *Hmgcr*. ChIP-seq data showed the peak on the promoter region of *Hmgcr* in WT sample. (B) *In vivo* binding of PEG3 to *Hmgcr* in the MEF cells. Individual ChIP experiment confirmed the binding of PEG3 to the promoter region of *Hmgcr* in MEF samples.
(TIF)

S4 Fig *In vivo* binding of PEG3 to the promoter of *Fasn*. ChIP -seq and individual ChIP experiments were performed to analyze the binding of PEG3 to *Fasn*. (A) Twenty-eight kb genomic regions surrounding *Fasn* from the ChIP-seq data. The peak on the promoter region of *Idh1* was observed in WT. (B) *In vivo* binding of PEG3 to *Idh1* in the MEF cells. Individual ChIP experiment confirmed the binding of PEG3 to the promoter region of *Idh1* in MEF samples.
(TIF)

S1 File This file contains the list of the downstream genes of *Peg3* in 30 metabolic pathways that were derived from ChIP-seq dataset. (XLSX)

S2 File This file contains the list of the RT and ChIP primers that were used for the qPCR and ChIP analyses. (XLSX)

S3 File This file contains the compiled raw data sets from qRT-PCR-based expression surveys. (XLSX)
