# Supplementary figures and images for: PEG3 controls lipogenesis through ACLY
Source: PLoS One. 2021 May 28;16(5):e0252354. doi: 10.1371/journal.pone.0252354 (PMC8162686; doi:10.1371/journal.pone.0252354)

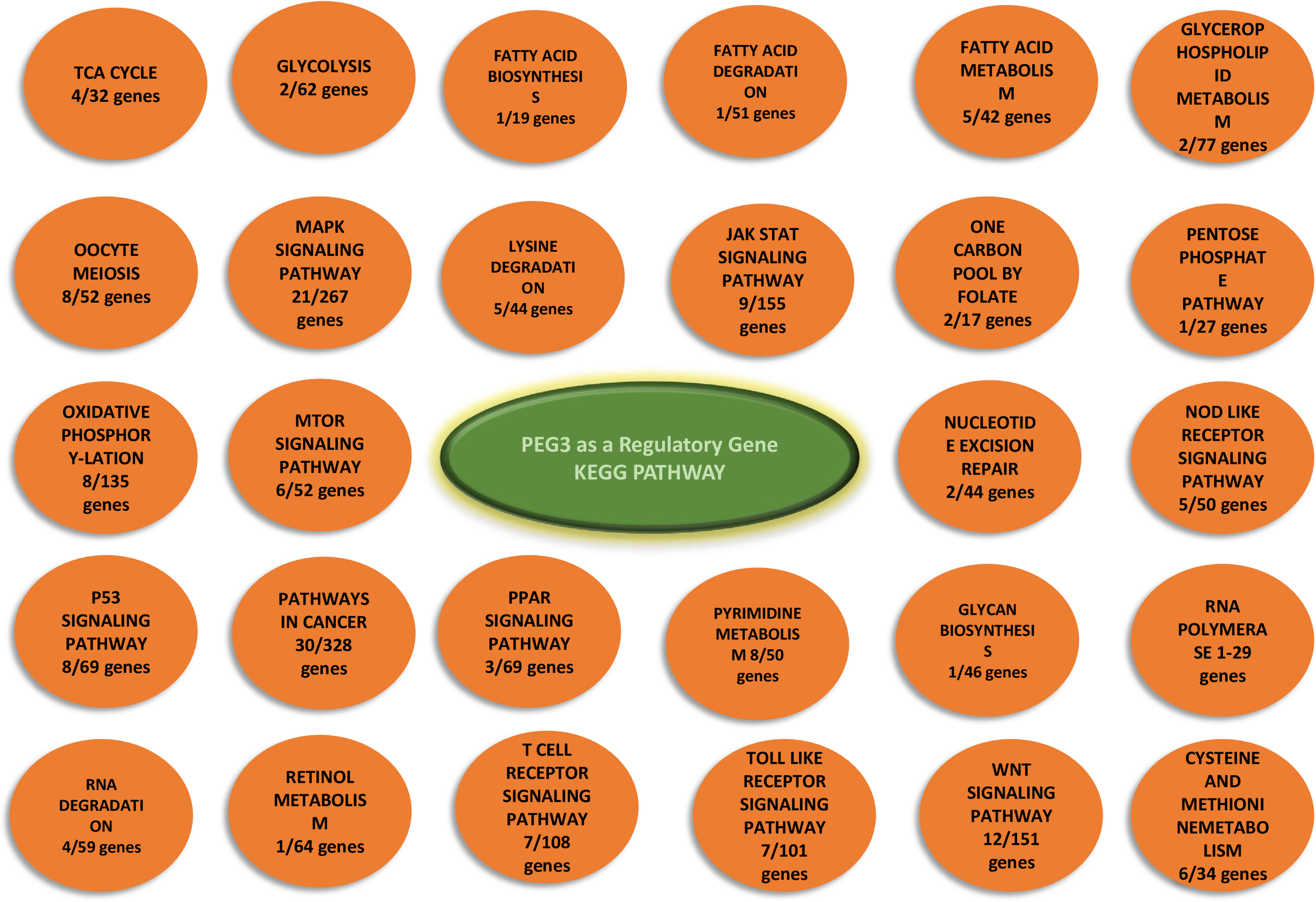

Supplement: S1 Fig — Thirty pathways were examined to find the overlapping genes between the gene list containing the downstream genes of Peg3 and the given pathway. Manual inspection of ChIP-seq results revealed 1073 downstream genes of Peg3. Out of these genes, several of them belong to already well-studied pathways as shown in the figure. (TIF) [file pone.0252354.s001.tif]

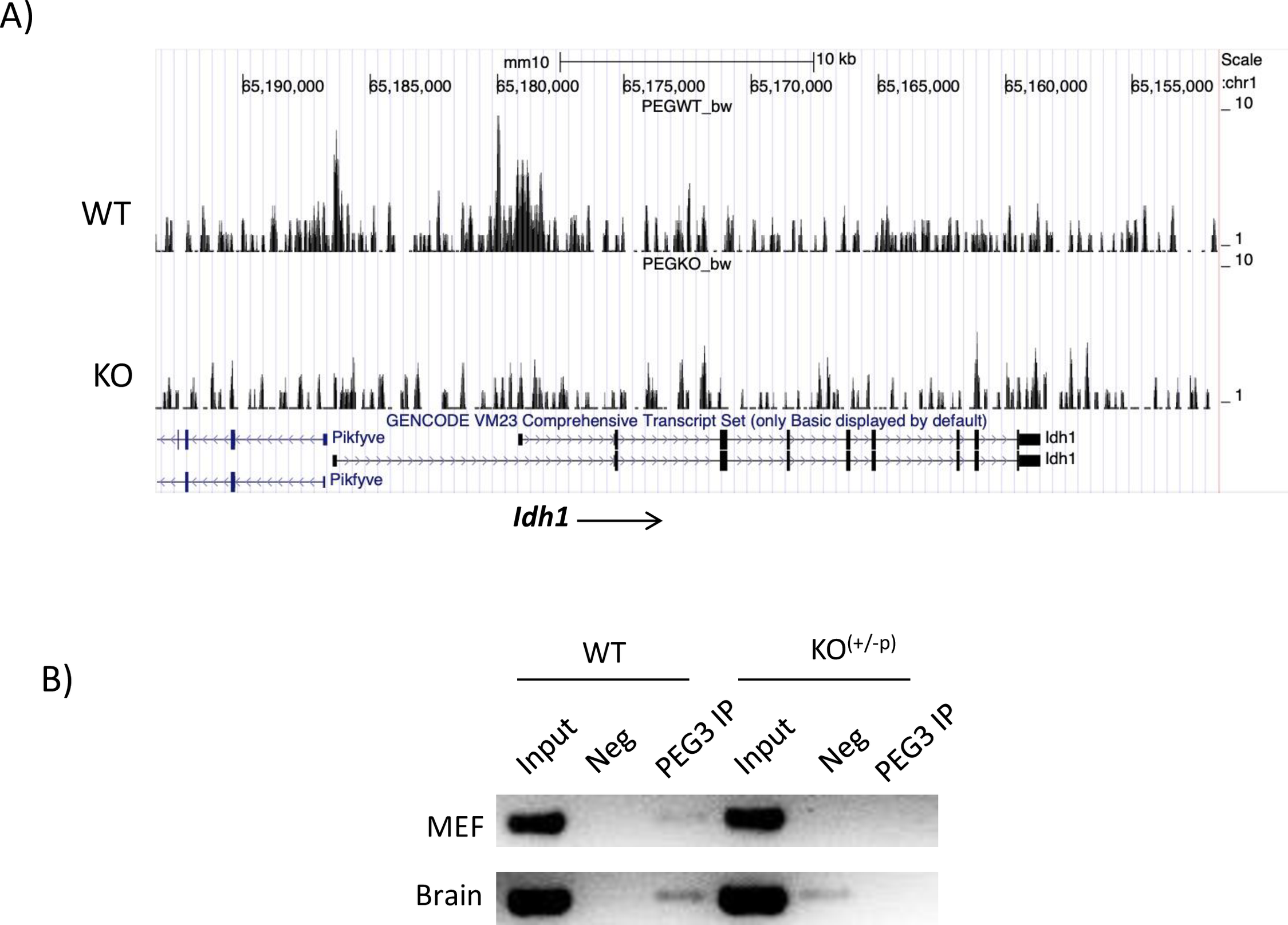

Supplement: S2 Fig — The binding of PEG3 to Idh1 was examined using ChIP-seq and individual ChIP experiments. (A) Forty-one kb genomic regions surrounding Idh1 from ChIP-seq data. The peak was observed on the promoter region of Idh1 in WT sample. (B) In vivo binding of PEG3 to Idh1 in the MEF and brain. Individual ChIP experiment confirmed the binding of PEG3 to the promoter region of Idh1 in both MEF and brain samples. (TIF) [file pone.0252354.s002.tif]

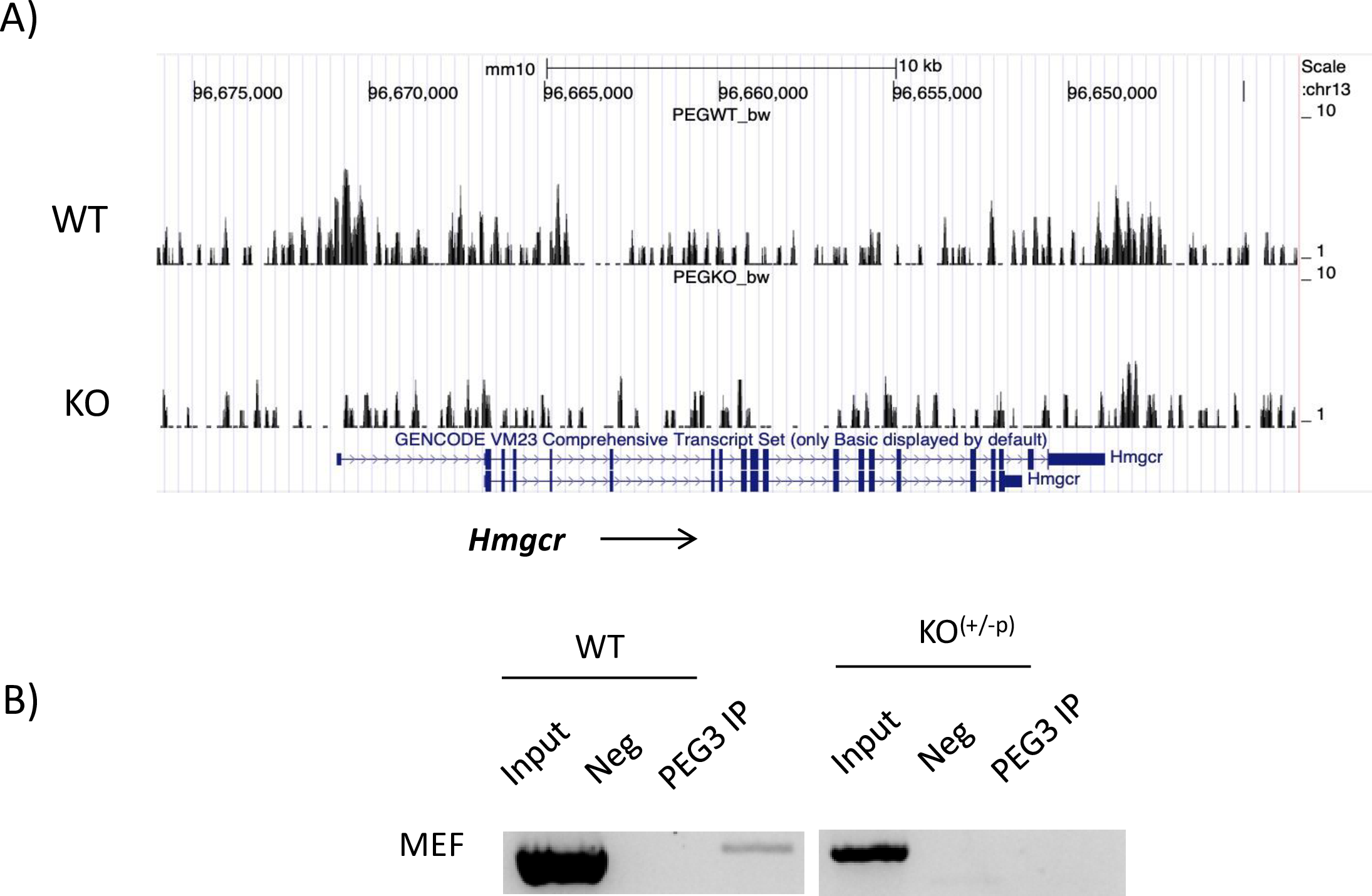

Supplement: S3 Fig — ChIP-seq and individual ChIP experiments were performed to assess the binding of PEG3 to Hmgcr. (A) Thirty-two kb genomic regions encompassing Hmgcr. ChIP-seq data showed the peak on the promoter region of Hmgcr in WT sample. (B) In vivo binding of PEG3 to Hmgcr in the MEF cells. Individual ChIP experiment confirmed the binding of PEG3 to the promoter region of Hmgcr in MEF samples. (TIF) [file pone.0252354.s003.tif]

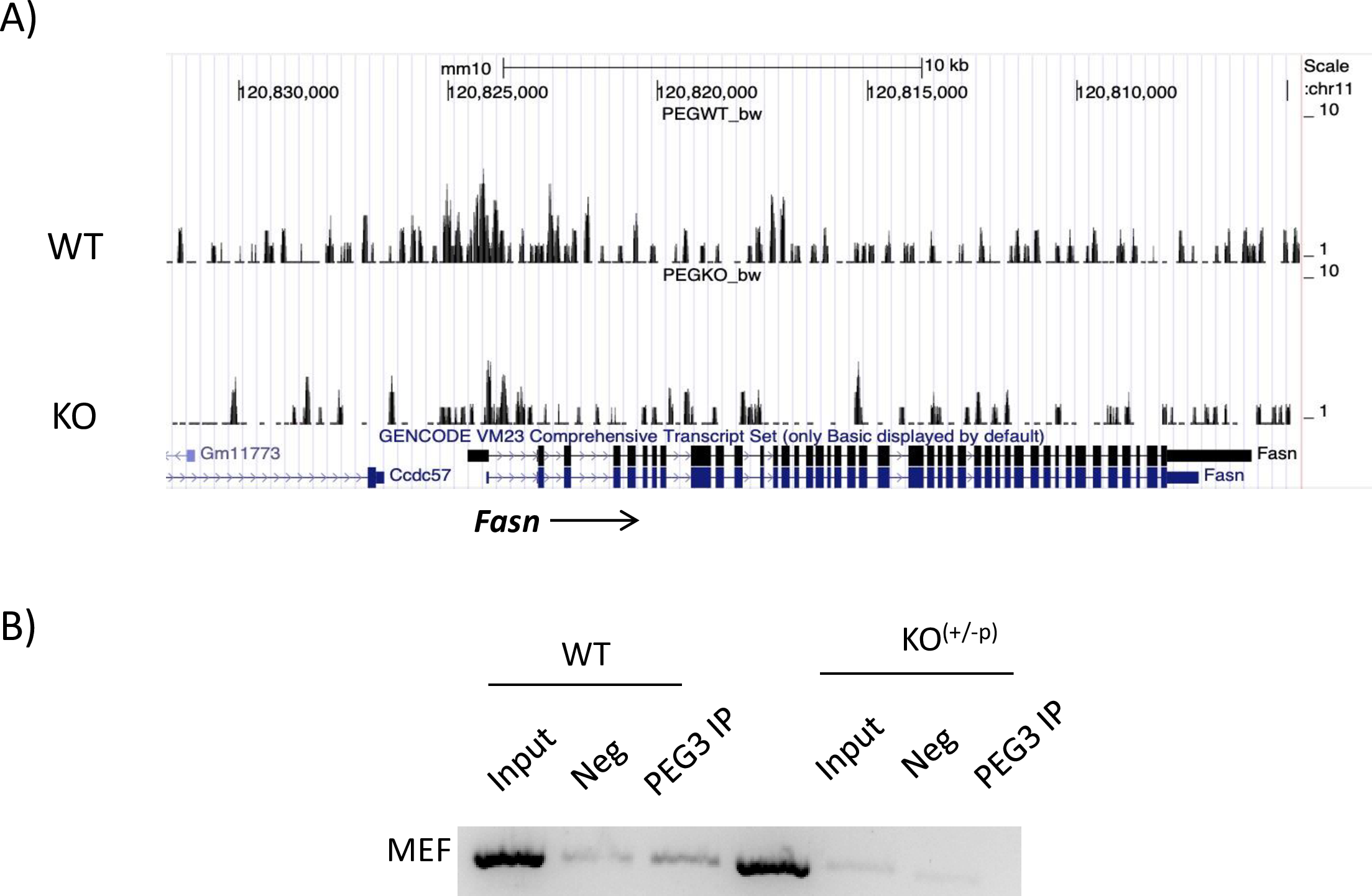

Supplement: S4 Fig — ChIP -seq and individual ChIP experiments were performed to analyze the binding of PEG3 to Fasn. (A) Twenty-eight kb genomic regions surrounding Fasn from the ChIP-seq data. The peak on the promoter region of Idh1 was observed in WT. (B) In vivo binding of PEG3 to Idh1 in the MEF cells. Individual ChIP experiment confirmed the binding of PEG3 to the promoter region of Idh1 in MEF samples. (TIF) [file pone.0252354.s004.tif]
